# Eleven-year follow-up of reconstruction with autogenous iliac bone graft and implant-supported fixed complete denture for severe maxillary atrophy: A case report

**DOI:** 10.1097/MD.0000000000018950

**Published:** 2020-02-28

**Authors:** Jae-Hyun Lee, In-Sung Luke Yeo

**Affiliations:** a Department of Prosthodontics, One-Stop Specialty Center, Seoul National University Dental Hospital; b Department of Prosthodontics, School of Dentistry and Dental Research Institute, Seoul National University, Seoul, South Korea.

**Keywords:** fixed complete denture, iliac bone graft, implant, long-term follow-up

## Abstract

**Rationale:**

The iliac bone graft procedure is one of the treatment options for individuals with extremely resorbed alveolar bones. An autogenous block bone graft can allow the use of an implant-supported fixed dental prosthesis, rather than conventional removable dentures, by completely edentulous patients. However, the iliac bone graft technique is an invasive procedure and should be carefully selected based on its long-term clinical results. This case report describes 11-year long-term outcomes for implant-supported complete denture on the grafted iliac bone.

**Patient concerns:**

A 68-year-old Asian man was referred for oral rehabilitation with fixed dental prostheses. The patient had been unsatisfied with his removable dental prostheses in masticatory performance.

**Diagnosis:**

Radiographical examination revealed severely atrophied maxilla.

**Interventions:**

The atrophied maxilla was reconstructed with an iliac block bone graft, after which an implant-supported fixed complete denture was placed.

**Outcomes:**

During 11 years of follow-up, several prosthetic and mechanical complications were encountered. Nevertheless, no biological complications were observed. Marginal bone levels around the implants were well-maintained on the radiographs after 11 years of prosthetic use.

**Lessons:**

Iliac bone graft can be chosen as a predictable treatment option that allows patients with extremely atrophic maxilla to use a fixed dental prosthesis instead of a removable denture.

## 1 Introduction

Maxillary removable complete dentures have been reported to have superior clinical outcomes to mandibular removable dentures.^[1,2]^ However, it is difficult to fabricate removable complete dentures for cases with excessive ridge resorption in the maxilla due to failed implants or combination syndrome.^[3–5]^ In these cases, implant-supported fixed dental prostheses can be an excellent treatment modality to restore the atrophic maxilla. Extensive alveolar bone reconstruction is, however, required because there is insufficient residual alveolar bone to place the implants.

Reconstruction of an atrophic maxilla is achieved using several techniques such as sinus augmentation and guided bone regeneration.^[6]^ Sufficient volume and height of alveolar bone is required for a successful long-term outcome of the implants.^[7]^ Iliac bone grafts have the advantage of high osteogenicity.^[8,9]^ The iliac bone graft uses autogenous bone, and these bone blocks have the ability to maintain space during bone formation.^[10,11]^ However, the procedure of necessity produces a second surgical site. Thus, using these grafts increases the number of scar-formation sites and the risk of infection and complications. Since the iliac bone graft procedure is invasive, it is necessary to confirm that this procedure can guarantee a good clinical outcome in the long term.

Numerous studies have reported that using an implant-supported fixed dental prosthesis in the maxilla has a high success rate.^[12–14]^ However, few studies have reported long-term clinical outcomes of implants placed on the iliac bone block grafted sites in an atrophic maxilla.^[15,16]^ This case report described the clinical outcome of an 11-year follow-up of implant-supported fixed complete denture placed on a maxilla reconstructed with an iliac bone graft.

### 1.1 Consent statement

The patient has provided informed consent for the publication of this case report and accompanying images.

## 2 Case report

**Figure 1:**
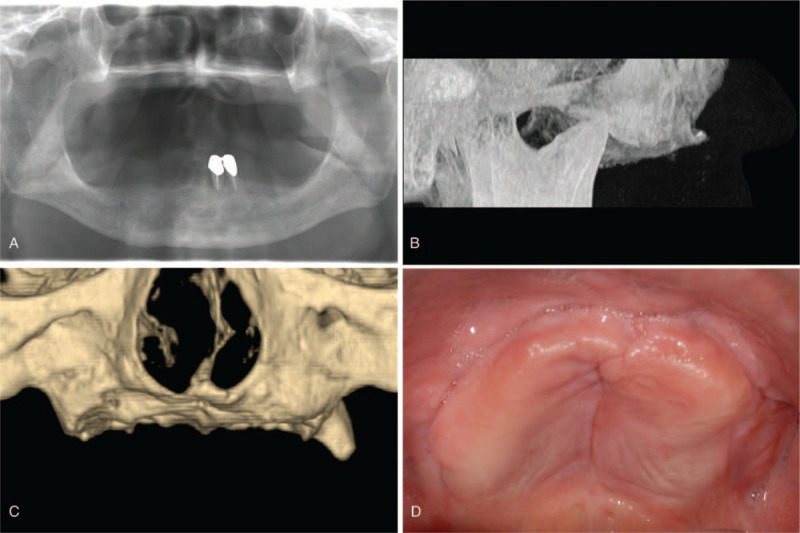
Pretreatment state. (A) Panoramic radiograph. (B) Lateral view in computed tomography. (C) Frontal view, reconstructed computed tomography image. (D) Occlusal view of the maxilla.

A 68-year-old Asian male patient, without significant systemic disease, was referred from a local dental clinic to the Department of Prosthodontics, Seoul National University Dental Hospital (Seoul, South Korea) in December 2006. The patient's chief complaint was “I am not satisfied with my removable dentures, especially while chewing food.” He had been wearing a maxillary removable complete denture and a mandibular removable partial denture. He had undergone removal of periosteal implants of the maxilla in the past. The patient did not have any systematic diseases (diabetes, osteoporosis, etc) or conditions (radiotherapy, bisphosphonate medication, etc). Clinical and radiographic evaluation revealed extremely resorbed alveolar bone on the entire maxilla and the posterior mandible. Computed tomography revealed little alveolar bone on the maxilla; only thin basal bone was left between the nasal cavity and the oral cavity. Flaccid tissue was found on the anterior area of the mandible, and the combination syndrome was suspected (Fig. 1).

After discussing multidisciplinary treatment options, it was decided to rehabilitate the patient's mouth with implant-supported fixed complete dentures on both the arches. The patient was referred to the Department of Oral and Maxillofacial Surgery for placement of autogenous onlay grafts to regain ridge height and width and permit implant placement. The anterior iliac crest of the left ilium was selected as the donor site.

General anesthesia was performed by an anesthesiologist. Oral and maxillofacial surgeons made a vestibular incision virtually over the whole of the maxillary arch. Mucoperiosteal flaps were elevated, and the pyriform aperture and the alveolar crest were exposed. Severe resorption of the alveolar bone was noted. A corticomedullary iliac bone block (60 × 40 mm) was taken from the left ilium, using a saw, chisel, and mallet, and was used to generate particulate marrow and cancellous bone (PMCB). Wound approximation was performed after bleeding control on the surgical field and H-vac insertion.

**Figure 2:**
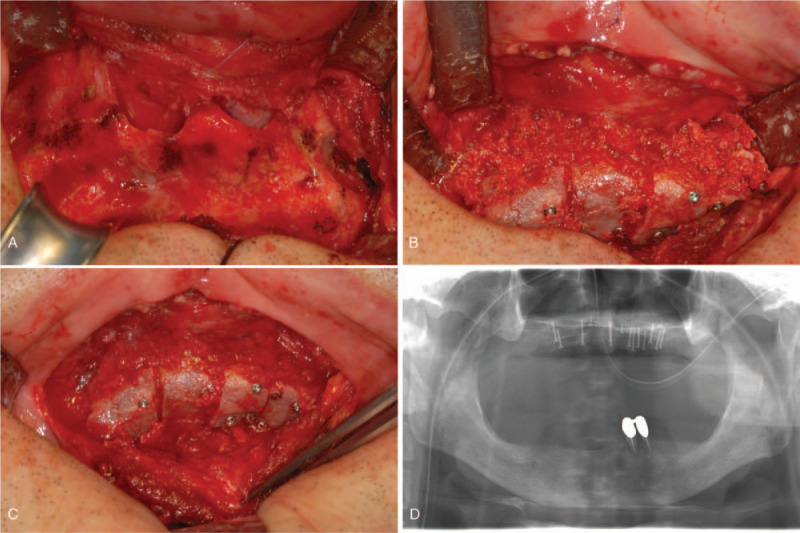
Iliac bone graft. (A) Severe atrophy of the maxilla seen after flap elevation. (B) Frontal view of iliac bone block graft with microscrews. (C) Occlusal view of iliac bone block graft with microscrews. (D) Panoramic radiograph after iliac bone graft.

The harvested bone was divided into 5 fragments and was laid on both the central incisal portion and the canine portion, using 4 fragments, while double layers were laid on the maxillary right canine area and fixed onto the basal bone with microscrews. The fragments were arranged in an arch. The gap between the bones was filled with the harvested PMCB, and fibrin glue (Tissucol/Tisseel, Baxter Healthcare, Deerfield, IL) was sprinkled on the grafted area (Fig. 2).

**Figure 3:**
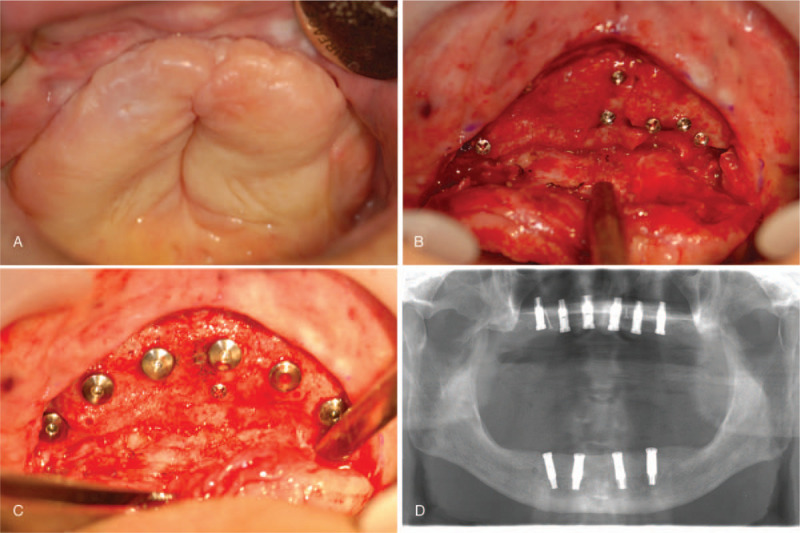
Implant surgery. (A) After 5 months’ healing. (B) After flap elevation. (C) Implant placement. (D) Panoramic radiograph after implant surgery.

After 5 months of healing, 6 bone-level dental implants (US II, external hex connection type, Osstem Implant Co., Busan, Korea) were placed in the maxilla (Fig. 3), and 4 implants (US II) were installed between both the mental foramens of the mandible. After allowing 3 months for osseointegration, the implants of the mandible were first uncovered and their healing abutments were connected. A provisional fixed complete denture of the mandible was fabricated to establish appropriate teeth arrangement, mandibular position, occlusal vertical dimension, and chewing pattern. After 3 months, the second-stage surgery was performed for placing the maxillary implants.

**Figure 4:**
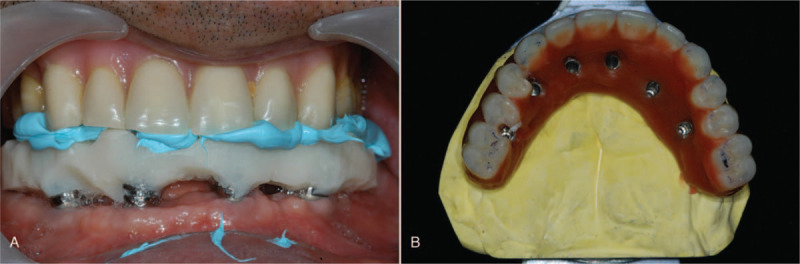
Prosthodontic treatment procedure. (A) Maxillo-mandibular relation registration. (B) Wax try-in denture.

**Figure 5:**
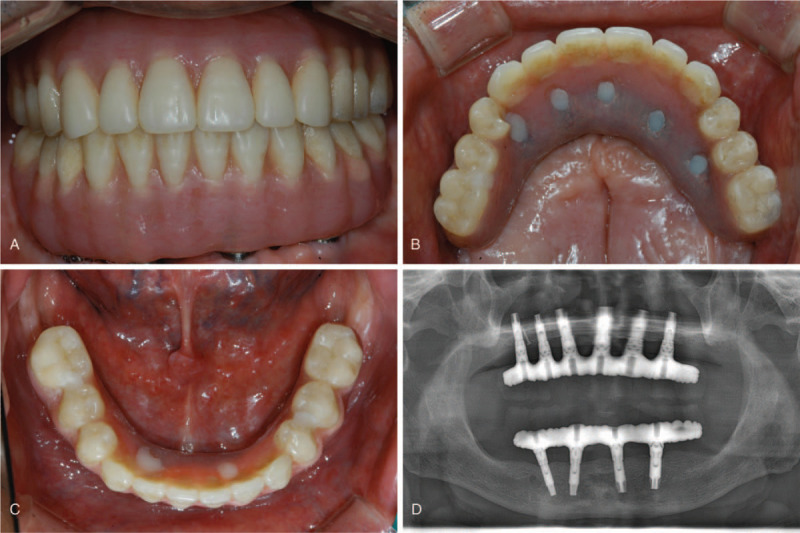
Definitive dental prosthesis. (A) Frontal view. (B) Occlusal view of the maxilla. (C) Occlusal view of mandible. (D) Panoramic radiograph after definitive prosthetic delivery (October 2008).

The abutments for the screw-type, implant-supported prosthesis (Esthetic-low abutment, Osstem Implant Co.) were tightened on all the implants. Definitive impressions were made at the level of the abutments using the open-tray impression technique, with pick-up impression copings. Polyvinylsiloxane impression material was used with custom trays fabricated from autopolymerizing acrylic resin (SR Ivoren, Ivoclar Vivadent, Schaan, Liechtenstein). The definitive casts were made using type IV dental stone (GC Fujirock EP, GC Europe N.V., Leuven, Belgium) and simulated gum material. Bite registration jigs were fabricated, and the maxillo-mandibular relationship of the patient was registered. A face-bow transfer was performed. Then, the master casts and the duplicate casts for the provisional prosthesis were cross-mounted onto a semi-adjustable articulator (Hanau Modular Articulator System, Whip Mix Corp., Louisville, KY). The milled titanium frameworks for the fixed complete dentures were fabricated for both arches. Wax-trial tooth arrangements were made on the frameworks and the wax dentures were evaluated intraorally to evaluate and confirm framework fit, lip support, mandibular position, and occlusion (Fig. 4). Subsequently, the definitive screw-type prostheses were fabricated by transferring the tooth arrangement and were then delivered intraorally. Prosthesis fit and occlusal function were reconfirmed. The prosthetic screws were tightened, and the screw-access holes were sealed in July 2008 (Fig. 5).

After delivery of the definitive prosthesis, it was used without any complications for 2 years, after which problems began to emerge. The artificial maxillary right lateral incisor and maxillary left canine had fractured. After replacement, the artificial teeth fractures recurred in the same area of the maxillary prosthesis 6 months later. The fractured sites were repaired again.

In the mandible, however, repeated screw fracture of the implants occurred after 3 years of follow-up. Six cases of prosthetic screw fractures and 1 case of abutment screw fracture of the mandibular fixed complete denture were observed in the course of about 1 year after 3 years of follow-up. It was considered that the mandibular 4-implant-supported fixed dental prosthesis could not withstand the excessive masticatory force of the male patient. Therefore, after approximately 4 years of follow-up (in April 2012), it was decided to replace the fixed mandibular prosthesis with a removable prosthesis, to reduce the occlusal load of the implants by distributing the load to the denture base-supporting soft tissue, while maintaining acceptable masticatory efficiency. Therefore, the hybrid prosthesis of the mandible was removed, and solitary stud-type attachments (Locator, Zest Anchors Inc., Escondido, CA) were connected to the placed implants to convert the superstructure into a 4-implant-retained removable complete denture.

During the next 7 years, there were no further complications of the mandibular denture. The maxillary implants had another 3 instances of prosthetic screw fractures and 2 artificial teeth fractures, but these could be easily repaired. The patient has been satisfied with his implant-supported prostheses in clinical performance.

**Figure 6:**
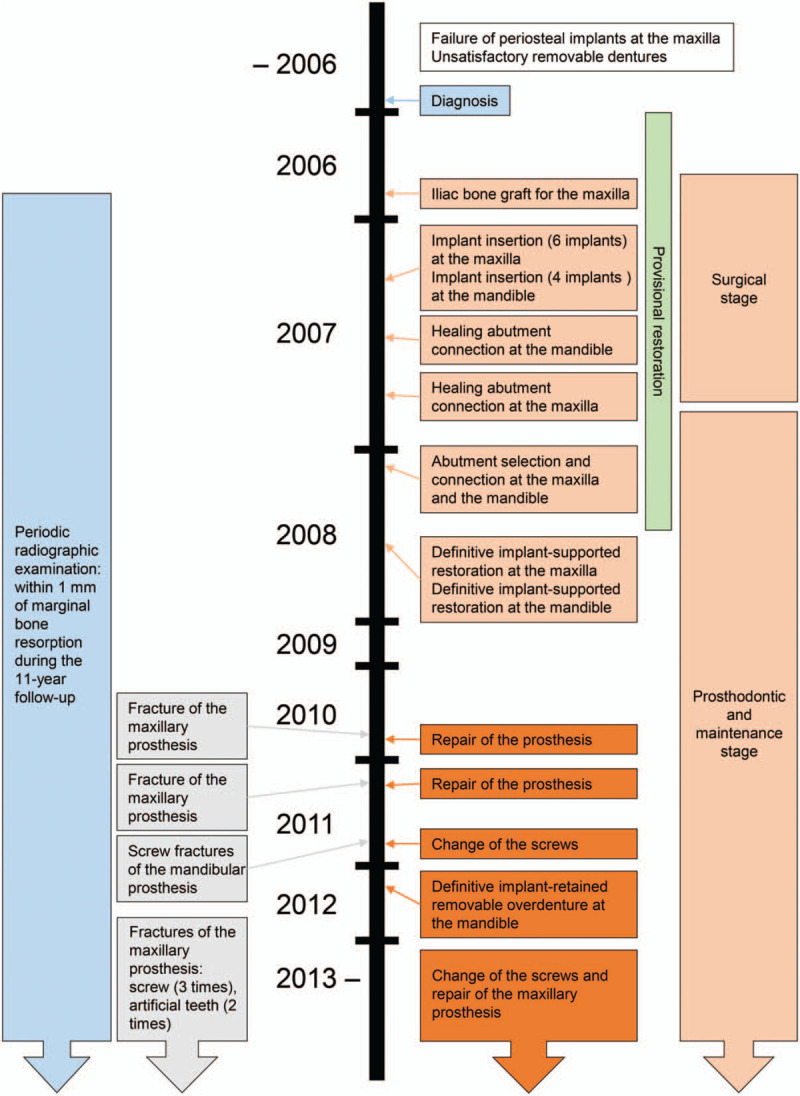
Timeline of interventions and outcomes.

**Figure 7:**
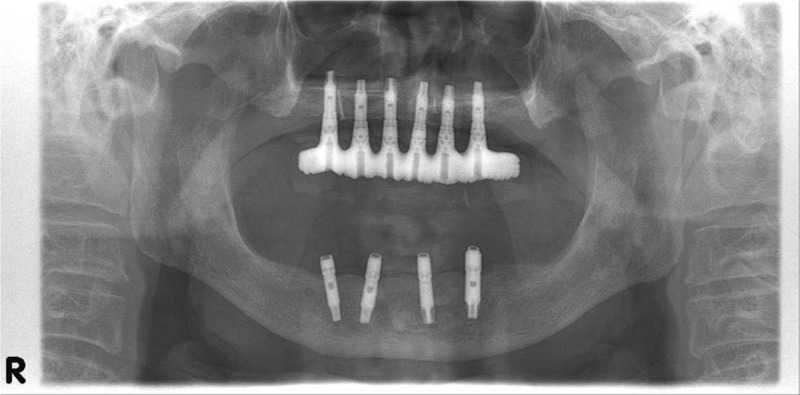
Panoramic radiograph after 11 years of follow-up (March 2019).

In summary, during the 11-year follow-up period after delivery of the maxillary and mandibular definitive prostheses, the mandibular fixed hybrid prosthesis was remade into a removable implant-retained overdenture, while the maxillary prosthesis, which was placed after an iliac bone graft, was well-managed and maintained with occurrence of only minor complications (Fig. 6). Little marginal bone loss, which was <1 mm during the observation period, was observed around any of the implants in a panoramic radiograph (Fig. 7).

## 3 Discussion

Complete arch fixed implant-supported prostheses afford life-changing benefits to fully edentulous patients.^[17]^ In this clinical study, use of an iliac bone graft resulted in fixed prosthetic rehabilitation of an atrophic edentulous maxilla and improved mastication and esthetics.

The anterior iliac crest is a commonly used donor site when both cortical and cancellous bone are needed.^[18]^ Indeed, iliac bone grafts provide vital bone support for implant placement without any antigen-antibody reaction.^[19]^ Conversely, complications may occur at the donor site. As the complication of iliac bone fracture has been reported in some cases, the procedure should be carefully selected.^[19]^ An implant survival rate of 98.6% during a mean follow-up of 7.9 years was recently reported for 10 patients who received an iliac bone graft in the maxilla and were then rehabilitated with complete-arch implant-supported hybrid prosthesis.^[20]^ However, no information was described for the status of the patients’ antagonistic arches in this previous study.^[20]^ The difference in masticatory force depending on mandibular restorative modalities may have a significant effect on the outcome of the maxillary treatment. In the present case report, mechanical complications continuously occurred when the opposed arch was a fixed hybrid prosthesis, but the complications decreased by replacing the opposed fixed dental prosthesis with a removable overdenture. However, some studies have reported that patient Oral Health-Related Quality of Life decreases with the use of removable prostheses,^[21–23]^ which should be taken into account when determining treatment plans.

The complete arch fixed complete dentures used in this study were fabricated with a titanium framework and used acrylic resin for artificial teeth and gingiva. This design remains popular because of its long track record in the literature, simplicity, reduced cost, easy reparability, and clinicians’ comfort level with using this material over the years. However, multiple clinical studies and systematic reviews have reported a high rate of prosthetic complications, such as fracture and wear of the acrylic resin and the need for repair, replacement, and lifelong maintenance.^[24–27]^ This maintenance represents a significant inconvenience for both the practitioner and the patient, in addition to financial costs.

Consistent with these reports,^[24–27]^ many prosthetic problems also occurred in this clinical case study. In the maxilla, the artificial resin teeth fractured repeatedly, although this problem was easily resolved. When the mandible was restored using the All-on-4 concept, the fixed prosthesis was eventually converted to an implant-retained removable denture after a series of multiple screw fractures, which was a prosthetic and mechanical complication. However, the marginal bone level around the mandibular implants was maintained well after 11 years of follow-up and no biological complications were observed. If biological complications, such as peri-implantitis or osseointegration failure, occur in addition to prosthetic complications, the patient would eventually experience loss of the implant-assisted prosthesis and return to the previous oral status of using conventional, removable complete dentures.

In the present case report, the maxillary marginal bone that was reconstructed using the iliac bone graft could be well-maintained because of the stress-relieving fractures of the prosthetic components. The prosthetic component fractures comprised minor complications that occurred first; therefore, excessive masticatory load or premature contact was not transmitted to the implants or the surrounding tissue.^[28]^

## 4 Conclusion

In this clinical case report, the maxillary alveolar bone reconstructed by extensive iliac bone block graft was well-maintained and functioned well during the 11 years of follow-up. Thus, iliac bone graft may be chosen as a predictable treatment option that allows the use of a fixed dental prosthesis instead of a removable denture in patients with extremely atrophic maxilla. When a patient receives a prosthesis after extensive bone grafts, a prosthodontic modality that is designed with consideration of masticatory stress and load distribution may be helpful, as illustrated in this case.

## Acknowledgment

The authors would like to express their sincere thanks and gratitude to Professor Jong-Ho Lee, Department of Oral and Maxillofacial Surgery, School of Dentistry, Seoul National University, for all surgical treatments.

## Author contributions

**Conceptualization:** Jae-Hyun Lee, In-Sung Luke Yeo.

**Data curation:** Jae-Hyun Lee, In-Sung Luke Yeo.

**Formal analysis:** Jae-Hyun Lee, In-Sung Luke Yeo.

**Investigation:** Jae-Hyun Lee, In-Sung Luke Yeo.

**Methodology:** Jae-Hyun Lee, In-Sung Luke Yeo.

**Project administration:** In-Sung Luke Yeo.

**Resources:** Jae-Hyun Lee, In-Sung Luke Yeo.

**Software:** Jae-Hyun Lee, In-Sung Luke Yeo.

**Supervision:** In-Sung Luke Yeo.

**Validation:** Jae-Hyun Lee, In-Sung Luke Yeo.

**Visualization:** Jae-Hyun Lee, In-Sung Luke Yeo.

**Writing – original draft:** Jae-Hyun Lee.

**Writing – review & editing:** Jae-Hyun Lee, In-Sung Luke Yeo.

Jae-Hyun Lee orcid: 0000-0002-2631-7722

In-Sung Luke Yeo orcid: 0000-0002-6780-2601
